# Orleans Parish Medical Society

**Published:** 1893-08

**Authors:** August McShane

**Affiliations:** Secretary; New Orleans, La.


### *SOCIETY REPORTS.*

---

### ORLEANS PARISH MEDICAL SOCIETY.

---

NEW ORLEANS, LA., April 29, 1893.

DR. A. J. BLOCH read a paper on

A CLINICAL REPORT OF A CASE OF HEPATIC ABSCESS.

(See page 337.)

DISCUSSION.

DR. DE ROALDES asked concerning the size of the cavity and the prognosis. Dr. Bloch said that the cavity was very large ; the patient was getting rapidly worse, and when first seen death seemed to be but a question of a few days. An operation was imperatively demanded.

DR. CHASSAIGNAC said that the thanks of the society were due to Dr. Bloch for a graphic report of his case. It was highly interesting, not only on account of its clinical features, but it possessed the rather unusual merit of reporting a fatal termination. Many writers unconsciously acquire the habit of not reporting their unfavorable cases. Dr. Bloch's case impresses one important fact—namely, that the patient is not entirely out of danger even when the pus escapes freely through the lung. He believes that if Dr. Bloch had continued to pack the cavity with iodoform gauze instead of injecting a liquid, the patient would have made an uneventful recovery. He does not see how a little coughing could tear open such firm adhesions as must have existed in a case of such long standing. He disagrees with Dr. Bloch, however, in attributing death to the escape of liquid into the peritoneal cavity. He cannot realize how the introduction of a comparatively small amount of warm water into the peritoneal cavity could give rise to fatal shock. In these days of abdominal surgery we drench the cavity with warm water without shock resulting.

Dr. Sexton said that an abscess of the liver may open in several different directions: It may discharge into the stomach, the intestines, through the diaphragm and the right lung, and it has been known to burst into the pericardium. It is not improbable that the injected liquid might have found its way into the pericardium in Dr. Bloch's case, for the heart stopped before the respiration. We need not be surprised at the fatal result in Dr. Bloch's case; most of his own cases had died. Those of his cases that recovered were usually superficial abscesses of the left lobe; the large abscesses of the right lobe were generally fatal. He has a case now under treatment in which the abscess was in the right lobe, and was quite large. He made an incision about four months ago, between the fifth and sixth ribs, and evacuated about two quarts of pus. He practiced irrigation, and passed a drainage tube through the opening. The cavity shrunk up so much that the tube could no longer be introduced. The man's wife has for some time performed the irrigating. The patient is now strong enough to come to the doctor's office. He has gained fifteen pounds in weight. Dr. Sexton condemns trephining of the ribs as a routine practice in abscess of the liver for the following reasons: necrosis of the exposed ends of the ribs is bound to take place, and thus a new disease is added to the old one. Furthermore, pus is apt to be absorbed through the cancellous tissue of the bone. Second, the traumatism incidental to the operation complicates the case. Third, the sharp ends of the rib after the operation act as an irritant to the surrounding soft parts. Dr. Sexton has now in his service in the Charity Hospital a man whose rib has been resected for empyema. The empyema is well, but the man wants to have the necrosis attended to. Where practicable, a free incision in an intercostal space is to be preferred to resection. Last year he had a case of superficial abscess of the left lobe that was working its way to the surface; the diagnosis was very easy. In order to help the pus to reach the surface, he directed the patient to lie always on his stomach. He did this for awhile, and his condition was all that could be desired; but he became tired of lying on his belly all the time, and he changed to the dorsal position. The pus infiltrated the deeper parts of the liver and the man died.

DR. DE ROALDES did not think that the injection of warm water into the peritoneal cavity could have caused death from shock. During the Franco-Prussian war, while he was a surgeon in the French army, Dr. Robert, now practicing in Pau, was abandoned at Amiens, after the battle of Sedan, as unable to continue in service on account of sickness. Subsequently his old teacher, Dr. Moutard-Martin, diagnosed a hepatic abscess. The abscess was opened with a free incision, and irrigation practiced. During the irrigations the doctor frequently spat up mouthfuls of injected liquid. Dr. de Roaldes saw Dr. Robert two years ago; he was then a healthy man, and did not at all look as though he had once been regarded as a hopeless invalid. In those days resection of the ribs had not become popularized. Altogether Dr. de Roaldes had treated about thirteen cases; of these only two recovered. One of the successful cases was a vegetable dealer of strong build. He resected one and one-half inches of rib, which afforded free drainage; but he afterward had to treat the necrosis of the exposed ends of the rib. In the other case the abscess was opened with the galvano-cautery, and simply irrigated. In another case, in consultation with Dr. Gaudet, the abscess was also opened with the galvano-cautery and the cavity packed with iodoform gauze. In a case that he saw with Dr. Renshaw, of this city, the abscess was opened and irrigation practiced. One day while they were irrigating, some liquid came out that had not gone in; the patient's wife recognized it as some broth that he had swallowed a few minutes before. He also vomited some carbolized water and had a diarrhœa of carbolized water. Later, a solution of some analine-dye was injected, and the man vomited some of the colored liquid, and had a colored diarrhœa.

Dr. Matas said that he regretted that he had arrived too late to hear Dr. Bloch's paper. The subjeet was one of unusual interest to him on account of the rather large number of cases that had come under his observation. When he stated at the last meeting of the State Medical Society, in 1892, that he had treated more than twenty-five cases since 1880, some surprise was caused by the statement. Dr. Matas was not able on that occasion to state the exact percentage of mortality or of recoveries because he had not yet tabulated and analyzed his cases, but he intended to do this fully in a future paper, and the results, he thought, would show a positive gain in the percentage of recoveries since the adoption of modern surgical methods. His ideas as to the prognosis of hepatic abscess had been positively changed for the better since his student days. When he was a resident student at the Charity Hospital thirteen years ago he had been impressed most unfavorably with the career of these cases, so that he had begun his practice with the conviction that the diagnosis of hepatic abscess was almost as bad as a patient's death warrant. The unfavorable results then were due in his opinion to the extreme conservatism of the times, which caused the practitioner to avoid an active surgical interference. The patients were treated in the medical wards and repeated aspiration with some modifications was the rule. The result was protracted hectic, increased loss of liver substance and final death from marasmus. Operation by incision, if performed, was always done late, after all other measures had been exhausted and the patient was reduced to the most unfavorable condition for true surgical interference.

He thought that he had met with all the classical and typical conditions of hepatic abscess and many others that were not typical. He had not met with an accident such as recorded by

Dr. Bloch. In abscess which had not burst into the bronchi he thought irrigation was certainly indicated and free from bad consequences. In the liver it was not as in the pleural cavity, where injection with even sterilized water had been followed by disastrous and even fatal consequences. He could not believe that death had been caused in Dr. Bloch's case by the escape of some of the injected fluid into the peritoneum, because the accidental escape of the hepatic pus of tropical abscesses without fatal results had been recorded; especially had this been noticed by the French, who, in their recent and extensive experience in Anam and Cochin China, had learned to practice the early evacuation of hepatic abscess by incision, without waiting for adhesions to form.

He, himself, made it a practice to wash out the abscess cavity thoroughly, and for this purpose always endeavored to gain free access to the interior of the cavity by free incision. He has at present in one of his wards in the Charity Hospital a case of enormous abscess of the right lobe of the liver, which illustrates his usual practice. In this case a free incision was made in the right hypochondrium, which allowed a good inspection of the interior of the cavity and revealed an anfractuous surface lined with partially detached and disintegrating sloughs. During irrigation, with a hot dilute solution of peroxide of hydrogen (a hot solution of common salt is used very frequently), the interior was swabbed and scrubbed carefully with a large mop of absorbent cotton or sterilized gauze held in the bite of a long hysterectomy forceps. This served the purpose of a safe blunt curette. It is necessary to do more than simply evacuate the pus; we must scrub away the partially detached masses of sphacelated tissue that line the cavity and which can never be expelled by simple irrigation through a tube, unless this be by a long and wasting suppuration. In this particular case, there was a long history of protracted hectic, and dysentery.

The patient was marasmic to an extreme degree. Dr. M. even hesitated as to the propriety of any operation. Still he felt it his duty to give the man a chance, and as he appeared to stand the anæsthetic (ether) better than was anticipated, he treated the abscess in the usual way. The right lobe reached the crest of the ilium, and over three quarts of pus escaped through the abdominal incision. After swabbing the interior with a cotton mop the cavity was packed thoroughly with iodoform gauze (5 per cent.) saturated with an emulsion of iodoform and glycerine 5 per cent. The external dressings consisted in a heavy layer of bichloride gauze covered with absorbent cotton and held in place by a broad roller-bandage. The patient rallied perfectly from the operation, much to the operator's surprise, but the dysentery returned, as frequently occurs in these cases, but was checked finally. Over three weeks have elapsed since the operation, and the abscess is becoming smaller every day; the appetite has returned; there has been no fever, but in the last twenty-four hours a diffuse lymphangitis of the right foot and leg has set in which it is feared will alter most unfavorably the patient's prospects. Dr. Matas had tried the new antiseptic, *alumnal*, in this case. He was not dissatisfied with the iodoform emulsion which he always used in systematically packing the cavity, but he thought that he had here an excellent case in which to test the reputed pus-inhibitory properties claimed for this agent. The alumnal was used in glycerine solution (5 per cent.), with which the cavity was freely irrigated before packing. These irrigations were made daily for a week, and were perfectly well borne by the patient. There was no irritation, and Mr. Lovell, the interne of the service, who conducted the after-treatment, was well satisfied with it, believing that the dressings were less soiled with its use than with solution; still it is difficult to estimate the comparative merits of a new agent in one case.

What Dr. Matas desired to emphasize most was the *early surgical* treatment of hepatic abscess, and by this he meant free incision, blunt curetting (scrubbing), irrigating, and thorough antiseptic packing. This is the ideal treatment applicable, because it converts an internal abscess into an open wound.

An incidental question is that of resection, or, rather, the recision of one or more ribs in order to gain free access to the cavity. This procedure should not be arbitrarily or unconditionally condemned. There are cases in which it is indicated, and others in which it is not. We should be guided by the condition of the abscess and of the patient. He recalled a case that came under his observation not long ago. The patient was a man from Honduras. He had a large dysenteric abscess of the right lobe, which pointed below the ribs. It was simply incised, irrigated, scrubbed and packed. One year after the same patient came with another abscess, but this time it involved the convexity of the liver, and Dr. Matas was compelled to resect three inches of the sixth rib in order to open a free avenue into the cavity. The patient again recovered, and he did not have necrosis of the exposed ends of the rib, nor has this been noticed in any of his cases. In another case, which he treated with Dr. Veazie, the liver retracted under the ribs after a simple incision in the hypochondrium; and in order to pack the cavity thoroughly he resected four inches of the right costal arch and thereby made an osteoplastic flap which allowed a free exposure of the hepatic cavity. Dr. Matas is opposed to trephining the ribs, because it is easy to injure the intercostal vessels and nerves, and because unnecessary traumatism is caused thereby without the compensatory advantage of a sufficient opening. The ribs should be excised subperiosteally with the periostotome. The soft parts may be easily peeled off from a rib by simply passing a strong strip of sterilized gauze under the rib and moving it backward and forward in the manner of a chain saw. The denuded rib may be excised with a costotome or bone-cutting forceps, or short saw, to any desired length.

Dr. M. J. Magruder did not think it always necessary to resect a rib even when the abscess had to be opened above the lower border of the ribs, and mentioned three case he had treated by simple free incision between the ribs, two of which made good recoveries, and the third is now under treatment and doing well. In two of these cases more than a pint of pus was evacuated, and in the other about ten ounces. In none of these cases was the cavity packed. Two large drainage tubes were introduced and the cavity irrigated daily with a warm 10 per cent. solution of hydrogen peroxide. Two of these cases occurred in the same person, who had a third one opened just below and an inch to the right of the ensiform cartilage. One week after the third abscess was opened there was a profuse discharge of bile, which continued several days, but diminished as the cavity filled up and finally ceased, the patient making a good recovery.

August McShane, M. D., Secretary.

